# Bone disease in early detected Gaucher Type I disease: A case report

**DOI:** 10.1002/jmd2.12314

**Published:** 2022-06-26

**Authors:** Vincenza Gragnaniello, Alessandro P. Burlina, Renzo Manara, Chiara Cazzorla, Laura Rubert, Daniela Gueraldi, Ermanno Toniolli, Emilio Quaia, Alberto B. Burlina

**Affiliations:** ^1^ Division of Inherited Metabolic Diseases, Department of Diagnostic Services University Hospital Padua Italy; ^2^ Neurology Unit St Bassiano Hospital Bassano del Grappa Italy; ^3^ Department of Neurosciences University Hospital Padua Italy; ^4^ Department of Cardiology and Radiology St Bassiano Hospital Bassano del Grappa Italy; ^5^ Department of Radiology University Hospital Padua Italy

**Keywords:** bone marrow infiltration, Gaucher disease, glucosylsphingosine, LysoGb1, newborn screening, osteonecrosis

## Abstract

Gaucher disease (GD) is a lysosomal disorder characterized by the storage of glucosylceramide in macrophages (“Gaucher cells”), particularly in the spleen, liver, and bone marrow. The most common phenotype, GD type 1, usually presents with hepatosplenomegaly, cytopenias, and sometimes bone involvement at variable age. Enzyme replacement therapy (ERT) is available and effective, but some severe manifestations are irreversible (e.g., osteonecrosis), so that early treatment is crucial. We describe a 4‐year‐old Albanian male with GD type 1, diagnosed through newborn screening (NBS), presented during follow up with multiple osteonecrotic areas in both femurs. He had no other symptoms or signs of disease, except for increasing of lyso‐Gb1 biomarker. Early initiation of ERT allowed a partial improvement of bone lesions. Our case highlights the importance of NBS for GD and of close follow‐up of presymptomatic patients, especially if biomarker levels are increasing. In the absence of NBS, GD should be considered in patients who present with bone lesions, also isolated. Early diagnosis and treatment improve the course of disease and avoid irreversible sequelae.

**Synopsis**

Isolated bone involvement can be an early manifestation of Gaucher disease type I.

## 1 INTRODUCTION

Gaucher disease (GD) is an autosomal recessive lysosomal storage disease resulting from deficiency of the β‐glucosidase (glucocerebrosidase) enzyme (*GBA1* gene), which hydrolyzes glucosylceramide (Gb1) into glucose and ceramide. It is a pan‐ethnic disorder affecting approximately 1:40000–1:60000 individuals worldwide. Glucosylceramide accumulates mainly in macrophages (“Gaucher cells”), particularly in the spleen, liver, and bone marrow; these cells play a fundamental role in GD pathogenesis.^1^

Traditionally, GD has been classified into three clinical phenotypes: Type 1, the non‐neuronopathic form, which is the most prevalent (94%); Type 2, the acute neuronopathic form, which is fatal in the first years of life (1%); Type 3, the chronic neuronopathic form, which has more gradual neurological involvement (5%).^2^, ^3^ The age at onset and clinical presentation of Type I GD is variable. Splenomegaly is observed in >90% of patients and may be the only clinical sign, but it is usually associated with hepatomegaly and cytopenias (thrombocytopenia, anemia, and rarely leukopenia). Children manifest growth retardation and delayed puberty. Because of the gradual onset, diagnosis is often delayed for several years. Over time, bone marrow infiltration causes osteopenia and skeletal symptoms, especially in the pelvis and lower limbs.^4^

Painful bone crises are probably associated with ischemic vaso‐occlusive phenomena. They may be reversible, but they usually cause bone infarcts and osteonecrosis (metaphyses or diaphyses of both long and flat bones) or avascular necrosis (epiphyses). The infiltration of Gaucher cells and abnormal production of cytokines also influence bone remodeling with loss of bone mass, cortical thinning, lytic lesions, fragility fractures and Erlenmeyer flask deformity of the femurs.^5^

MRI is the method of choice to evaluate the skeleton and bone marrow in GD,^6^ despite the need for sedation in younger children and the fact that assessment of infiltration is more difficult due to the presence of red marrow in the long bones.^1^, ^4^

Other manifestations, such as lung and renal involvement, hematological or solid neoplasias, and extrapyramidal disease, are rare.^4^

The initial diagnosis of GD is based on measurement of acid β‐glucosidase activity levels in total white cells, mononuclear cells, fibroblasts, or dried blood spots (DBS), and is confirmed by molecular analysis.^1^, ^7^, ^8^

Treatment is typically administered to GD Type 1 patients who have signs and symptoms^1^, ^9^ and includes enzyme replacement therapy (ERT) (imiglucerase, velaglucerase, taliglucerase, 30–60 U/kg EOW) or substrate reduction therapy (miglustat or eliglustat, only for adults). Enzyme replacement improves hematological, visceral, and, over time, bone manifestations, but some abnormalities are irreversible (e.g., avascular necrosis and bone infarction sequelae), so that early diagnosis and treatment are important to prevent permanent complications.^4^, ^9^

Here, we report a pediatric patient with GD Type 1 identified through NBS who manifested femur osteonecrosis as the first sign of the disease. Early ERT allowed an improvement of bone abnormalities, preventing severe irreversible sequelae.

### 1.1 Case report

Our patient is an Albanian male 4 years 8 months of age, affected by GD Type 1. At birth, NBS revealed reduced β‐glucosidase activity (0.88 uM/L, nv >4.2) and elevated lyso‐Gb1 values in DBS (77.35 nmol/L, nv 5.64–33.31). Molecular analysis of the *GBA1* gene revealed compound heterozygosity: c.1448 T > C (p.Leu483Pro) + c.1226A > G (p.Asn409Ser) (L444P and N370S according to previous nomenclature, respectively). This genotype suggests a non‐neuropathic disease (due to a N370S allele) but the presence of the L444P mutation tends to increase disease severity.^10^ Plasma lysoGb1 was also elevated (17.65 nmol/L, nv 1–12.3).

**FIGURE 1:**
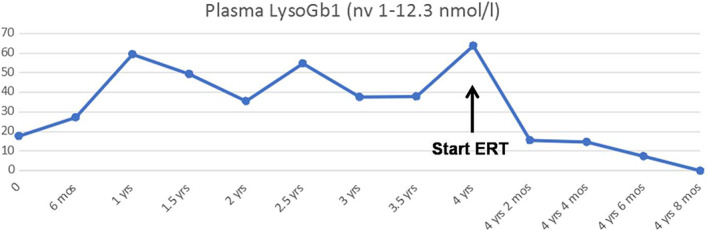
Plasma lyso‐Gb1 trend: LysoGb1 levels progressively increase during the first years of life, and then rapidly decrease after initiation of enzyme replacement therapy (ERT).

**FIGURE 2:**
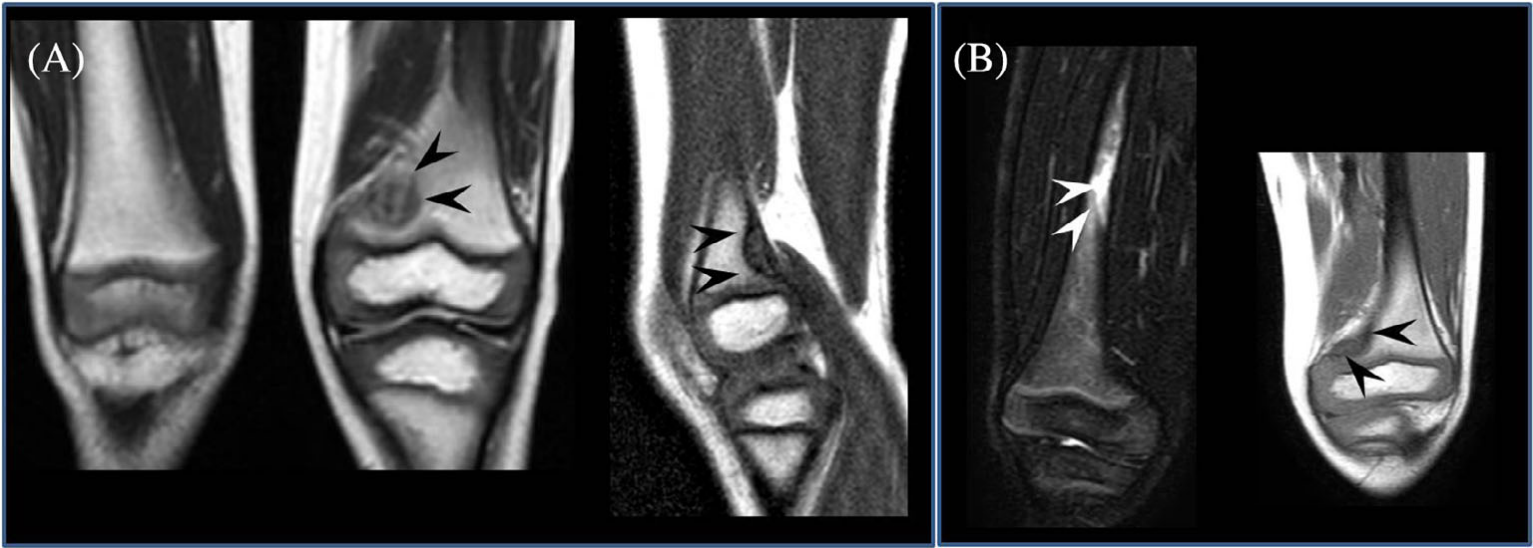
MRI of the femurs before the start of therapy (A) hypointense lesion in T1‐weighted sequences at the meta‐diaphyseal region of the left femur (12 × 4 × 13 mm) also with evidence of sclerotic border (black arrows). (B) 3 months after the first MRI, time 0 at the start of ERT: appearance of confluent infarction lesions hyperintense in T2‐weighted sequences (total max diameter 3 cm) in the central area of the diaphysis of right femur (white arrows). Stable hypointense lesion in T1‐weighted sequences in the left femur (black arrows).

**FIGURE 3:**
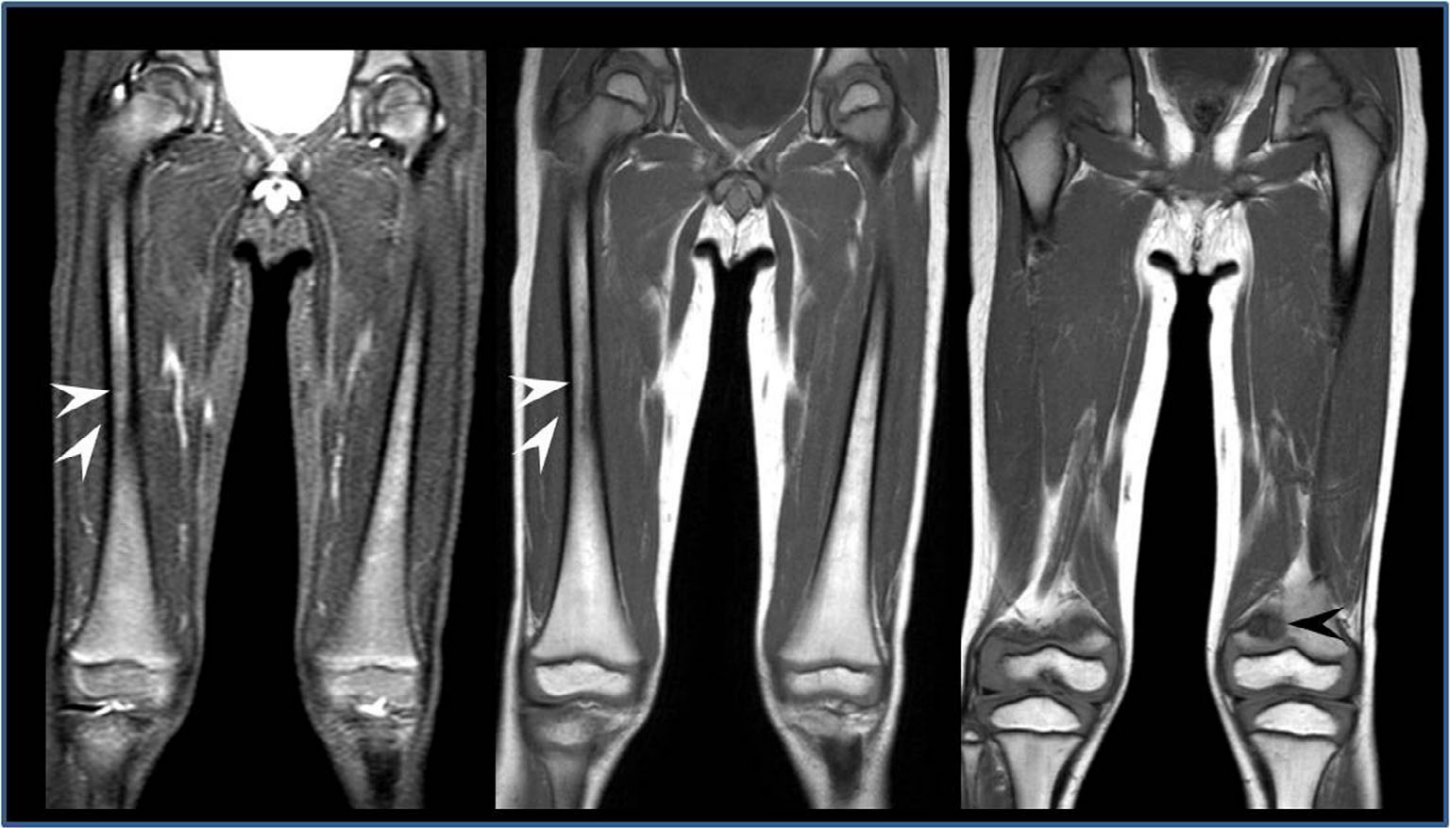
Radiological follow up after 8 months of ERT. Improvement of the lesions in the right femur (white arrows), stable lesion in the left femur (black arrows). Lesions appear hypointense in T1‐weighted sequences.

At first evaluation, he had no symptoms or signs of GD, so he started follow‐up (clinical, biochemical and instrumental [abdominal echography]) every 6 months, presenting regular psychomotor development and no signs or symptoms of GD. He never experienced hepatosplenomegaly, cytopenias, or other biochemical abnormalities, except for a progressive increase in plasma lyso‐Gb1 (Figure 1). At the age of 3 years 8 months, lyso‐Gb1 levels was increased up to 63.5 nmol/L (nv 1–12.3). There is a lack of detailed guidelines for the follow up of pediatric patients with GD diagnosed by newborn screening (NBS). Irreversible bone complications are known to occur in patients with the L444P genotype and, although the patient did not report bone pain, we decided to perform a baseline femur MRI. This revealed bone marrow infiltration, and an area of osteonecrosis at the meta‐diaphyseal region of the left femur (Figure 2A). Two smaller similar lesions were found at the epiphysis of the left femur and at the proximal region of the diaphysis of the left tibia. X‐rays were negative. After 3 months, an MRI also showed new confluent infarction lesions (max. Diameter 3 cm) in the central area of the diaphysis of right femur (Figure 2B). The patient did not report pain, in contrast with the severity of bone lesions. Laboratory tests were normal, including complete blood count and liver enzymes. Lumbar and femoral Dual Energy X‐ray Absorptiometry scans were also normal. However, plasma lyso‐Gb1 remained elevated (63.75 nmol/L, nv 1–12.3 nmol/L). Because of the rapidly progressive bone lesions, the patient started ERT with imiglucerase 60 U/Kg EOW at 4 years of age, resulting in rapid improvement of the lyso‐Gb1 biomarker (Figure 1). After 8 months of therapy, he does not present any new signs or symptoms of GD. Femoral MRI shows a reduction of infarction lesions on the right femur and a stable osteonecrotic area on the left femur (Figure 3). No new lesions have appeared.

## 2 DISCUSSION

Bone involvement in patients with GD is not rare, although it tends to appear later in the disease course than visceral manifestations. Due to frequent diagnostic delays, skeletal manifestations can be present at diagnosis, but rarely are they isolated manifestations. Rossi et al. analyzed a cohort of 44 patients with pediatric‐onset GD and demonstrated that bone involvement was the revealing clinical sign in only 32.4% of patients (about half of them with radiologic evidence of avascular necrosis, bone infarction, lytic lesions, or pathological fractures). Instead, the main signs at diagnosis are usually splenomegaly (96.9%) with or without hepatomegaly (60.8%), anemia (53.3%), and/or thrombocytopenia (86.7%). Patients with bone symptoms at presentation were significantly older than those with only organomegaly (9.8 vs. 5.6 years), confirming the later involvement of bone.^11^

Data from 887 children included in the International GD Registry also reveals that 94% of pediatric patients have visceral and/or hematological involvement at the time of diagnosis.^12^ Therefore, current diagnostic algorithms are based on well‐recognized hematological and visceral manifestations,^9^, ^13^, ^14^ while isolated bone symptoms are not usually suspected in association with GD at clinical presentation.

There are only a few reported cases of GD presenting with isolated bone manifestations in children. In 1999, Graber et al. described a 12‐year‐old female with relapsing hip arthritis without splenomegaly as the first manifestation of GD.^15^ In 2009, Kennouche et al. reported an 11‐year‐old male who presented isolated unilateral osteochondritis of the femoral head and, subsequently, osteomyelitis of a knee with negative culture. GD was suspected from the finding of “Gaucher cells” on bone biopsy and was confirmed with enzymatic and molecular tests.^16^ Recently, Olivieri et al. designed a diagnostic algorithm (BIG, Bone Involvement in GD) that considers bone symptoms to facilitate early GD diagnosis. They identified two female children ages 7 and 8.5 years with bone involvement (aseptic osteomyelitis and recurrent lower limb pain, respectively) as initial manifestations of GD without cytopenias or significantly visceromegaly.^17^ Finally, Chis et al. described an 11‐year female patient with right shoulder pain due to a humeral cyst. Other osteolytic lesions were found in the skull. Her skeletal symptoms were predominant, but not isolated, as she also had a slightly enlarged spleen and liver that pointed to the GD diagnosis.^18^

Despite that all these patients presented with disabling symptoms, they experienced significant diagnostic delays. Notably, our patient presented at 4 years of age with severe radiologic bone involvement as the first isolated manifestation of GD. This early diagnosis of bone involvement highlights the role of NBS and may support the benefits of early intervention in the disease.

Due to the patient's genotype, the progressive increase in lysoGb1 levels, the progression of radiological lesions (3 months of follow up) and the lack of guidelines in very early diagnosed patients, we decided, in agreement with the family, to start ERT. After 8 months of therapy, the patient showed improvement of the lesions of the right femur (more recent) and stability of the lesions of the left femur. Although MRI is invasive and requires sedation in pediatric patients, bone MRI can be very important in the follow up of presymptomatic children carrying severe genotypes. However, further studies on large populations are needed to establish the correct management of these patients and to analyze the advantages of early diagnosis over the risk of over medicalization.

In our Region (Northeast Italy), NBS for LSD, including GD, has been performed since 2015 by measuring enzyme activities with MS/MS.^19^, ^20^ Several diagnostic technologies are now available (e.g., DMF, MS/MS)^21^, ^22^; however, NBS for GD is still controversial and is not included in the Recommended Uniform Screening Panel in the US.^23^ The controversy stems from ethical considerations related to the diagnosis of later‐onset forms.^20^ Our case demonstrated that NBS is important to identify the first signs of the disease and start early treatment, avoiding the common diagnostic delay and severe irreversible lesions. Recently, a Delphi consensus also supported NBS for GD, so that its inclusion in NBS should be considered.^9^

Nevertheless, further efforts are likely to be required to fully satisfy the criteria for adding GD in the neonatal programs of screening. The demand for routine monitoring and appropriate treatment are still debated for GD NBS.^1^, ^9^ The frequency of monitoring could be based on genotype,^1^ but the phenotype is not always perfectly predicted.^24^ Therefore, biomarkers play a fundamental role. A recent systematic review^25^ and a large study in a pediatric population^26^ demonstrated that lyso‐Gb1 is the most reliable biomarker currently available for diagnosis, prognosis, and can be used for treatment monitoring of patients with GD. Excessive bone levels of lyso‐Gb1 have been shown to directly mediate osteoblast dysfunction.^27^ Lyso‐Gb1 can be measured in plasma and DBS,^28^, ^29^ and is already elevated at birth in GD and increases over time. In the era of NBS, it has the potential to reduce the false positive rate as a second‐tier test and, despite not indicating the need of ERT if isolated, trends can inform decision‐making regarding follow‐up frequency and treatment initiation.^9^, ^20^, ^25^

In our patient, increasing lyso‐Gb1 levels were useful to establish a close follow‐up that included femurs MRI, despite the need for sedation. It is also a reliable biomarker of response, preceding changes in other disease parameters. Of note, our patient's lyso‐Gb1 levels are lower than those usually found in clearly symptomatic patients, but long‐term follow‐up studies are needed in patients diagnosed by neonatal screening for a better interpretation of the use of lysoGb1 as a biomarker for monitoring the disease.

## 3 CONCLUSIONS

The clinical suspicion of GD Type 1 is traditionally associated with splenomegaly, hepatomegaly and cytopenia, and diagnostic algorithms have been developed based on these signs. We present a pediatric patient with GD in which isolated bone involvement was the first clinical manifestation. The early disease recognition through NBS and close follow‐up allowed early initiation of ERT, preventing the progression of bone disease. Further studies on large populations are needed to establish the correct management of these patients and to analyze the advantages of early diagnosis over the risk of over medicalization. In the future, specific guidelines for the follow‐up and the start of therapy in presymptomatic patients should be developed.

Moreover, our case highlights that bone involvement in GD may not reflect the disease in other organs, with progressive and severe skeletal disease occurring in patients who have little or no visceral and hematologic involvement. This observation has 2 implications. First, in presymptomatic patients diagnosed through NBS or family screening, skeletal monitoring could be useful, regardless of involvement of other systems. Moreover, if NBS is not performed, it is important to include GD in the diagnostic algorithm in cases of bone signs or symptoms, also if isolated and in patients with incidental radiological lesions. This could avoid diagnostic and therapeutic delays and the development of severe and irreversible sequelae.

## AUTHOR CONTRIBUTIONS

**Vincenza Gragnaniello:** conceptualization, methodology, investigation, resources, patient's management, data curation, writing‐original draft, visualization; **Alessandro** **P.** **Burlina:** conceptualization, investigation, resources, data curation, writing‐review; **Renzo** **Manara:** investigation, resources, MRI studies, data curation, writing‐review; **Chiara** **Cazzorla:** resources, patient's management; **Laura** **Rubert:** methodology, resources, patient's management; **Daniela** **Gueraldi:** methodology, resources, patient's management; **Ermanno** **Toniolli:** resources, MRI studies; **Emilio** **Quaia:** resources, MRI studies; **Alberto B.** **Burlina:** conceptualization, methodology, investigation, resources, patient's management, data curation, writing‐original draft and review, visualization, supervision, funding acquisition.

## FUNDING INFORMATION

The authors reported there is no funding associated with the work featured in this article.

## CONFLICT OF INTEREST

Vincenza Gragnaniello, Alessandro P. Burlina, Renzo Manara, Chiara Cazzorla, Laura Rubert, Daniela Gueraldi, Ermanno Toniolli, Emilio Quaia, Alberto B. Burlina declare no conflict of interest.

## INFORMED CONSENT STATEMENT

All procedures followed were in accordance with the ethical standards of the responsible committee on human experimentation (institutional and national) and with Helsinki Declaration of 1975, as revised in 2000. Informed consent was obtained from parents of the subject involved in the study. Proof that informed consent was obtained is available upon request.

## Data Availability

Data available on request due to privacy/ethical restrictions.
